# Measuring the relative contribution to predictive power of modern nucleotide substitution modeling approaches

**DOI:** 10.1093/bioadv/vbad091

**Published:** 2023-07-14

**Authors:** Thomas Bujaki, Katharine Van Looyen, Nicolas Rodrigue

**Affiliations:** Department of Biology, Carleton University, Ontario K1S 5B6, Canada; Institute of Biochemistry, Carleton University, Ontario K1S 5B6, Canada; Department of Biology, Carleton University, Ontario K1S 5B6, Canada; Department of Biology, Carleton University, Ontario K1S 5B6, Canada; Institute of Biochemistry, Carleton University, Ontario K1S 5B6, Canada; School of Mathematics and Statistics, Carleton University, Ontario K1S 5B6, Canada

## Abstract

Traditional approaches to probabilistic phylogenetic inference have relied on information-theoretic criteria to select among a relatively small set of substitution models. These model selection criteria have recently been called into question when applied to richer models, including models that invoke mixtures of nucleotide frequency profiles. At the nucleotide level, we are therefore left without a clear picture of mixture models’ contribution to overall predictive power relative to other modeling approaches. Here, we utilize a Bayesian cross-validation method to directly measure the predictive performance of a wide range of nucleotide substitution models. We compare the relative contributions of free nucleotide exchangeability parameters, gamma-distributed rates across sites, and mixtures of nucleotide frequencies with both finite and infinite mixture frameworks. We find that the most important contributor to a model’s predictive power is the use of a sufficiently rich mixture of nucleotide frequencies. These results suggest that mixture models should be given greater consideration in nucleotide-level phylogenetic inference.

## 1 Introduction

Markovian models of nucleotide substitution have now been in development for over 50 years. Starting from the first simple idea of Jukes *et al.* (1969), modeling substitution events as a Poisson process, this history has been one where the assumptions of existing models are progressively relaxed in order to better capture the features of molecular evolution when conducting phylogenetic inferences. While many innovations have been proposed, practical and computational limitations have constrained the set of models that are widely used to those of the *general time reversible* (GTR) family (Tavaré 1986). The GTR model is parameterized by two distinct sets of parameters: four nucleotide frequency parameters (three degrees of freedom), denoted as $\pi ={\left(\pi_{i}\right)}_{\left(1\leq i\leq 4\right)}$ and summing to 1, as well as six nucleotide exchangeability parameters (5 degrees of freedom), denoted as $\rho ={\left(\rho_{ij}\right)}_{\left(1\leq i,j\leq 4\right)}$. Together, these parameters specify the rates of the Markov generator governing the nucleotide substitution process along the branches of the phylogeny, denoted as $Q_{ij}=\rho_{ij}\pi_{j}$, which is to say that the instantaneous rate from nucleotide *i* to nucleotide *j* is given as the product of the relative exchangeability of nucleotides *i* and *j* and the frequency of the target nucleotide *j*. Special cases of the GTR model can be obtained by constraining either set of parameters, or both sets; for instance, the F81 model (Felsenstein 1981) is obtained from setting all *ρ_ij_* values to 1, and the JC model (Jukes *et al.* 1969) can be obtained by further setting all *π_i_* values to 1/4.

Choosing which model from this family to apply to a given dataset was a common concern under the small sample sizes of the early days of probabilistic phylogenetic inference. Thanks to the availability of practical tools (e.g. Posada and Crandall 1998), a common protocol was to select one of the special cases of the GTR model, based on information-theoretic criteria such as the AIC (Akaike 1974) or BIC (Schwarz 1978), and then conduct the phylogenetic inference *per se* utilizing the chosen model. By the turn of the century, however, these and other model selection methods began to elect the GTR substitution model as the most warranted for nearly all datasets (Sumner *et al.* 2012), which incorporated more and more sequences, of greater length.

Along the way, the importance of accounting for the heterogeneity in overall rates across sites became well understood, thanks to the gamma distribution approach of Yang (1993, 1994). The general idea behind the gamma-distributed rates model is to consider that different sites of the alignment have different branch length multipliers, i.e. positive real values that average 1 across sites; loosely speaking, branch lengths seem shorter if the multiplier is less than 1, and longer if it is greater than 1. The distribution across sites of these branch length multipliers follows a gamma law, constrained to a mean of 1, and with a variance given by $\alpha^{-1}$, with *α* itself considered a free parameter of the inference. The likelihood function then takes the form of an integral over the gamma law, usually approximated by discretization into a sum over four categories of rates. The gamma-distributed rates-across-sites model is also now nearly universally utilized on modern datasets.

Recent research now often also accounts for *pattern* heterogeneity in the substitution process. Rather than invoking a single GTR model, the basic idea to capturing pattern heterogeneity is to invoke several, either through a fixed-effect preset allocation of models to sites of the alignment (i.e. partitioning methods, Lanfear *et al.* 2012), or through a random-effects mixture modeling strategy (e.g. Pagel and Meade 2004). Within the random-effects mixture approaches, one considers that the sites of a multiple sequence alignment were generated with several different *Q* matrices. The number of *Q* matrices, as well as the proportion of sites considered to have been generated by each, is generally unknown. This constitutes another model selection challenge.

The potential richness of these partitioning and mixture approaches is a departure from the models typically considered during the development of selection criteria such as the AIC and BIC, which were comparatively low dimensional. With high-dimensional models, these information criteria have been shown to decrease in reliability (e.g. Yang and Barron 1998, Broman and Speed 2002, Brewer *et al.* 2016, Liu *et al.* 2023). Such findings may partly explain some of the conflicting results in studying partitioning methods based on these criteria (e.g. Brown and Lemmon 2007, Ho and Lanfear 2010, Cameron *et al.* 2012, Leavitt *et al.* 2013, Kainer and Lanfear 2015). In contrast, recent work by Lartillot (2023) has shown that some Bayesian cross-validation methods, such as the *sitewise* cross-validation method, can be reliable even for high-dimensional mixture models.

Here, we apply the sitewise Bayesian cross-validation score to compare a large set of nucleotide substitution models. Our objective is to quantify the relative importance of modeling three aspects of nucleic acid sequence evolution. First, we measure the importance of accounting for uneven exchangeabilities across the six possible pairs of nucleotides, by contrasting the simple F81 model (Felsenstein 1981) and the GTR model. Second, we quantify the importance of modeling the heterogeneity of overall rates, by contrasting a model with homogeneous rates against the gamma-distributed rates model of Yang (1994). Third, we evaluate the importance of capturing pattern heterogeneity, by contrasting single-matrix models against mixture models. The mixture models we consider have components that are distinguished by their own nucleotide frequency parameters, while all other parameters are common across components. Whereas nucleotide-level mixture models of this type have usually been limited to finite mixtures of only a few components (typically between 2 and 4), we explore finite mixtures up to 100 components, as well as infinite mixtures based on the Dirichlet process. These models are the nucleotide level versions of the CAT models at the amino acid level (Lartillot and Philippe 2004). We also explore every combination of all three modeling approaches, to assess potential synergizes or redundancies.

## 2 Materials and methods

### 2.1 Data

Four multigene datasets were studied here:

- Broughton: A concatenation of 20 nucleotide alignments of protein coding DNA (18 180 sites in total) from 61 species of fish, obtained from Broughton *et al.* (2013).
- Lartillot: A concatenation of 20 nucleotide alignments of protein coding DNA (15 117 sites in total) from 78 placental mammals, obtained from Lartillot and Delsuc (2012).
- Regier: A concatenation of 62 nuclear protein-coding genes (41 976 sites in total) from 75 arthropod species, and five species of tardigrades and onychophorans, obtained from Regier *et al.* (2010).
- Wainwright: A concatenation of 10 nuclear loci (8439 sites in total) from a sample of 188 acanthomorph (spiny-rayed fish) species, obtained from Wainwright *et al.* (2012).

We also studied four single-gene datasets:

- PB2: An alignment of 401 sequences of the influenza PB2 gene, 2277 nucleotides in length, taken from Tamuri *et al.* (2009).
- RAG1: An alignment of 88 sequences of the RAG1 gene sampled across tetrapods, 2613 nucleotides in length, taken from Hugall *et al.* (2007).
- RBCL: An alignment of 179 sequences of the RBCL (rubisco) gene sampled across eudicots, 1341 nucleotides in length, taken from Parto and Lartillot (2018).
- RHOD: An alignment of 38 sequences of the rhodopsin gene sampled across vertebrates, 900 nucleotides in length, taken from Yokoyama *et al.* (2008).

These datasets were chosen because of their high-quality, completeness, and open-access availability.

### 2.2 Models

We explore the following models:

- F81: The simplest model considered, built from a single set of nucleotide frequency parameters (Felsenstein 1981).
- GTR: A model built from a single set of nucleotide frequency parameters and pairwise nucleotide exchangeability parameters (Tavaré 1986).
- CAT-Poisson: A model built from a Dirichlet process on nucleotide frequency parameters, capturing pattern heterogeneity across sites, but without invoking nucleotide exchangeability parameters (Lartillot and Philippe 2004); the model can be viewed as an infinite mixture of F81 models.
- CAT-GTR: As before, a model built from a Dirichlet process on nucleotide frequency parameters, along with a set of nucleotide exchangeability parameters (Lartillot and Philippe 2004).
- CAT${}_{f=x}$-Poisson: A finite mixture version of CAT-Poisson with *x* components. We considered versions ranging from CAT${}_{f=2}$-Poisson (i.e. CAT${}_{f=1}$-Poisson amounts to the F81 model) to CAT${}_{f=100}$-Poisson. The number of components we explore includes every integer up to 10, then every 10 until reaching 100.
- CAT${}_{f=x}$-GTR: A finite mixture version of CAT-GTR with *x* components (from 2 to 100, with CAT${}_{f=1}$-GTR being the GTR model), along with a set of nucleotide exchangeability parameters.
- F81 + Γ: The F81 model with gamma-distributed rates across sites, discretized into four categories of rates (Yang 1994).
- GTR + Γ: The GTR model with gamma-distributed rates across sites; this model serves as the reference in our overall model ranking.
- CAT-Poisson + Γ: The CAT-Poisson model with gamma-distributed rates across sites.
- CAT-GTR + Γ: The CAT-GTR model with gamma-distributed rates across sites.
- CAT${}_{f=x}$-Poisson + Γ: The CAT${}_{f=x}$-Poisson with gamma-distributed rates across sites.
- CAT${}_{f=x}$-GTR + Γ: The CAT${}_{f=x}$-GTR model with gamma-distributed rates across sites.

To our knowledge, the CAT-inspired models have never been studied at the nucleotide level of the present work. We used the default priors in PhyloBayes (Lartillot *et al.* 2009) on all models.

### 2.3 Bayesian cross-validation

We carried out 5-fold cross-validation protocol on each multiple sequence alignment (Bujaki and Rodrigue 2022). The protocol is as follows:

1. Subdivide the dataset into a *testing set*, made from one fifth of the alignment columns drawn at random, and a *learning set*, made from the remaining four fifths of the alignment columns.
2. For a given model, a Bayesian Markov chain Monte Carlo algorithm is used to sample 1500 draws from the posterior distribution on parameters, conditional on the learning set; discarding the first 500 cycles as burn-in leaves a sample of *K* = 1000 draws from the posterior distribution. A particular set of parameter values from this sample is denoted *θ_k_*, where 1≤k≤K.
3. Writing the likelihood score at site *n* of the testing set as $p(D_{n}|\theta_{k})$, the site cross-validation score is taken as the average site likelihood over the entire sample of 1000 draws, and the overall sitewise cross-validation score is a sum of the natural logarithm of the site cross-validation score, written as:
$$\text{CV–score}=\sum_{n}\ln\left(\frac{1}{K}\sum_{k}p(D_{n}|\theta_{k})\right).$$
4. Repeat steps 2 and 3 for all models.
5. Using the GTR+Γ model as a reference, we calculate the difference cv-score between it and a model of interest (although any model can be used a reference).
6. Repeat the entire protocol five times, reporting the mean and standard deviation across the five.

We used PhyloBayes (Lartillot *et al.* 2009) for all calculations.

## 3 Results and discussion

### 3.1 Finite and infinite mixtures

**Figure 1.**
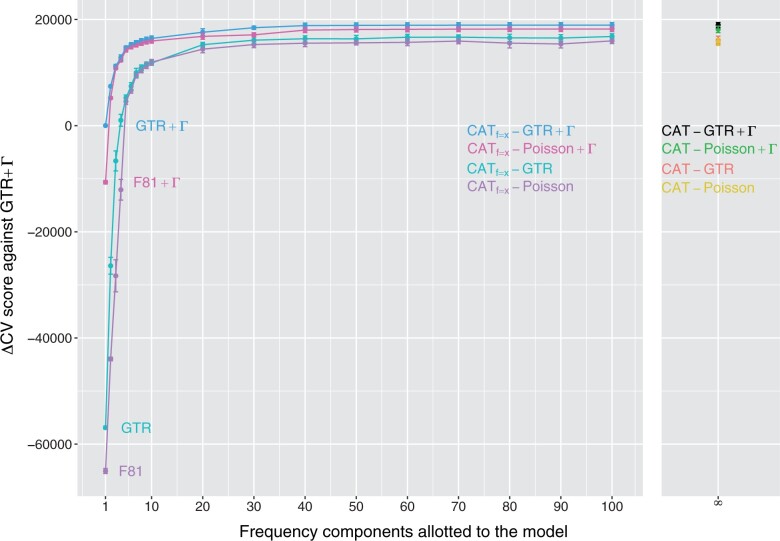
Cross-validation scores (relative to the GTR + Γ model) for the Regier dataset, plotted as a function of the number of nucleotide frequency components; note that models consisting of special cases with a single component are labeled near *x *=* *1.

We first ran our model comparisons on multigene datasets. Figure 1 shows the cross-validation results relative to the GTR + Γ model for a multigene dataset taken from Regier *et al.* (2010). The graph highlights how the finite mixture models’ scores climb rapidly with an increasing number of components. The cross-validation score of finite mixtures appears to plateau by around 30 components; increasing the number of components beyond this point does not increase the cross-validation score. Interestingly, a model built solely on a mixture of nucleotide frequency parameters (i.e. with even exchangeability parameters and homogeneous rates across sites) outperforms the reference GTR + Γ model. The best-performing models are those that combine a sufficiently rich mixture with free exchangeability parameters as well as gamma-distributed rates.

The variation across cross-validation replicates does not allow for a clear distinction between the rich finite mixture models and the infinite (CAT) models. In other words, for rich mixtures, the sampling error induced from splitting the original data into learning and testing sets (see Methods) leads to average cross-validation scores that are within each other’s error margins. These models are thus very similar in overall performance.

Table 1 shows that these results generalize across multigene datasets (reporting results for 100 component finite mixtures). From a practical standpoint, it may be simplest to utilize the infinite mixture model (CAT), rather than repeatedly applying finite mixtures with progressively more components until reaching a plateau, given that it automatically adapts to the heterogeneity within the data. These numbers can be interpreted as follows: Using the Regier data and CAT-GTR +Γ model compared to GTR +Γ, the per site cv-score is 18 941/41 976 = 0.451, making the average data column $e^{0.451}=1.57$ times better explained by CAT-GTR +Γ.

**Table 1.** Cross-validation score relative the GTR + Γ with multigene datasets.

	Broughton	Lartillot	Regier	Wainwright
F81	−8411.8 ± 189.3	−6475.1 ± 111.9	−65 060.6 ± 474.7	−11 609.6 ± 457.7
GTR	−6521.3 ± 139.6	−3796.8 ± 132.8	−56 885.8 ± 277.9	−8841.2 ± 285.2
CAT${}_{f=100}$ Poisson	1172.3 ± 32.8	283.0 ± 36.2	15 963.4 ± 503.6	1681.1 ± 61.6
CAT${}_{f=100}$ GTR	1397.1 ± 30.5	585.5 ± 41.6	16 791.6 ± 544.7	1859.6 ± 64.0
CAT-Poisson	1160.8 ± 46.9	269.5 ± 56.7	15 742.2 ± 643.8	1668.2 ± 57.2
CAT-GTR	1384.7 ± 45.4	585.4 ± 41.4	16 115.6 ± 741.7	1860.1 ± 63.5
F81+Γ	−1882.0 ± 80.8	−2782.7 ± 62.3	−10 656.4 ± 327.6	−2923.5 ± 246.6
CAT${}_{f=100}$ Poisson + Γ	1503.5 ± 35.1	561.2 ± 39.1	18 203.6 ± 480.7	1961.6 ± 60.0
CAT${}_{f=100}$ GTR + Γ	1777.9 ± 37.4	887.1 ± 38.7	18 935.4 ± 475.6	2211.9 ± 54.5
CAT-Poisson + Γ	1503.9 ± 34.1	291.3 ± 510.1	18 122.0 ± 612.8	1956.5 ± 61.8
CAT-GTR + Γ	1777.5 ± 35.7	889.0 ± 38.6	18 941.0 ± 475.3	2211.9 ± 54.2

### 3.2 Single matrix models

Unsurprisingly, the GTR model always outperforms the F81 model, across all datasets. Given the size of the multigene datasets (see Methods), the six (5 degrees of freedom) nucleotide exchangeability parameters can be reliably inferred, and it is well understood that nucleotides of any given pair are not evenly exchangeable as assumed by the F81 model. We have not explored other forms of single matrix models, given that the relative difference between F81 and GTR is modest when either of them is compared to the mixtures models we consider here.

### 3.3 Gamma-distributed rates across sites

Also well established is the fact that the gamma-distributed rates model always outperforms its homogeneous counterpart. Indeed, this modeling strategy introduces a single new parameter controlling the shape of the unit-mean gamma distribution, which can be reliably inferred from even single-gene datasets. It is also fully expected that positions across the alignments we study will exhibit variation in their overall substitution rates given variable pressures of selection under which they have evolved.

### 3.4 Contrasting modeling approaches

Still focusing on multigene datasets, Table 2 shows the difference in cross-validation scores between modeling approaches, rather than all against GTR + Γ. Reporting results in this way highlights the relative contribution of each approach to predictive power; while it is well-established that each of the modeling approaches reported above improve model performance, we still lack quantitative measurements of which of these makes the greatest contribution to model performance.

**Table 2.** Pairwise cross-validation score differences with multigene datasets.

Model difference	Models	Broughton	Lartillot	Regier	Wainwright
Mixture	F81 versus CAT-Poisson	9572.5	6744.6	80 802.8	13 277.8
Mixture	GTR versus CAT-GTR	7906.0	4382.3	73 001.4	10 701.3
Mixture	F81+Γ versus CAT-Poisson + Γ	3386.0	3074.0	28 778.4	4879.9
Mixture	GTR + Γ versus CAT-GTR + Γ	1777.5	889.0	18 941.0	2211.9
Γ distributed rates	GTR versus GTR + Γ	6521.3	3796.8	56 885.8	8841.2
Γ distributed rates	F81 versus F81+Γ	6529.7	3692.4	54 404.2	8686.1
Γ distributed rates	CAT-GTR versus CAT-GTR + Γ	392.8	303.6	2825.4	351.8
Γ distributed rates	CAT-Poisson versus CAT-Poisson + Γ	343.1	21.8	2379.8	288.2
Free exchangeabilities	F81+Γ versus GTR + Γ	1882.1	2782.7	10 656.4	2923.5
Free exchangeabilities	F81 versus GTR	1890.4	2678.3	8174.8	2768.4
Free exchangeabilities	CAT-Poisson + Γ versus CAT-GTR + Γ	273.5	597.7	819.0	255.5
Free exchangeabilities	CAT-Poisson versus CAT-GTR	223.9	315.9	373.4	191.9

The mixture modeling approach consistently leads to the largest difference in cross-validation score relative to its single-matrix counterpart. Contrasting the F81 model, which has a single nucleotide frequency vector as its parameterization, against the CAT model, which is based on an infinite mixture of nucleotide frequency vectors, shows the largest improvement in cross-validation score brought about by modeling pattern heterogeneity. When contrasting GTR with CAT-GTR, we still see a large improvement in cross-validation score, albeit not quite as large as F81 versus CAT. This suggests that there may be a slight overlap between the GTR and CAT modeling approaches; indeed a mixture of nucleotide frequency parameters can have the effect of modulating pairwise exchanges between nucleotides. Comparing F81+Γ against CAT + Γ still show a large improvement, although it is less than the F81 versus CAT comparison. This again suggests that the modeling approaches have a level of overlap; a mixture of nucleotide frequency parameters can induce variations in overall rates across sites. An example of this would be having nucleotide frequency vectors that are dominated by a single nucleotide, which would lead to low overall rates of substitution, and other frequency vectors that are even across the four nucleotides, which would lead to comparatively higher overall rates of substitution. The mixture modeling approach produces the smallest (though still relatively high) improvement in model performance in the GTR + Γ versus CAT-GTR + Γ context.

Table 2 also shows that the gamma-distributed rates-across-sites approach is the second biggest contributor to a model’s performance. Its contribution is of a lesser magnitude when coupled to a richer model, as expected from the partial overlap in capturing rate heterogeneity from the different modeling approaches. This is also the case for the GTR modeling approach, which yields the least improvement in performance of the three approaches. Some of the smallest increases in cross-validation scores are seen in the CAT + Γ versus CAT-GTR + Γ comparisons, as well as CAT versus CAT-GTR.

### 3.5 Single-gene alignments

**Figure 2.**
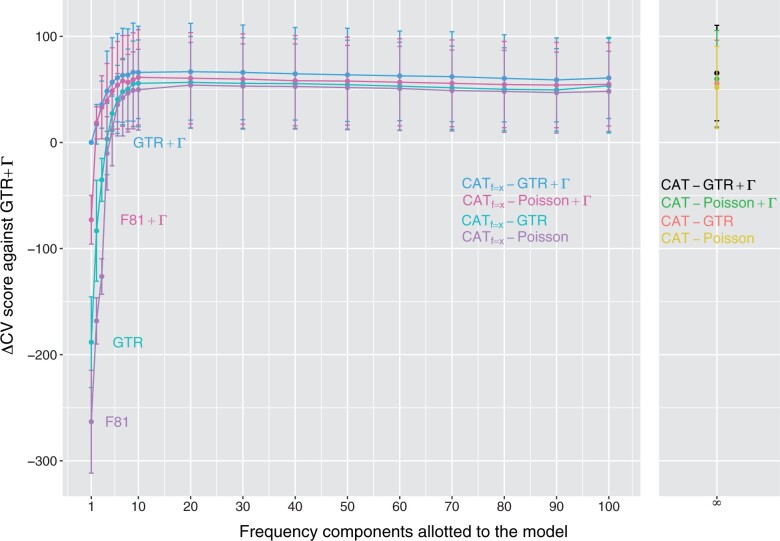
Cross-validation scores (relative to the GTR + Γ model) for the RBCL dataset, plotted as a function of the number of nucleotide frequency components; note that models consisting of special cases with a single component are labeled near *x *=* *1.

In order to study the relative model performance on smaller datasets, we performed the same analyses on single-gene alignments. Figure 2 shows the results of the cross-validation model comparisons with the RBCL gene. Overall, the same general trends observed with multigene datasets are found in the single-gene context. However, the 5-fold cross-validation protocol leads to a relatively higher variance in scores across the five replicates. This can be seen from the error bars that are much larger relative to the difference in cross-validation score than for results on multigene datasets. This is to be expected with smaller, single-gene alignments. Beyond the small size of the datasets, the error bars across the five cross-validation replicates can be attributed to even smaller learning sets (i.e. even less evolutionary signal from which to learn parameter values), and very small testing sets.

Another notable feature of Fig. 2 is the slightly decreasing trend in cross-validation scores for finite mixture models beyond 20 components. Although the error bars are large, preventing us from clearly distinguishing between these different mixture models, we believe this reflects the intrinsic penalty induced by the cross-validation protocol: over-parameterized models lead to a decrease in predictive power, with parameter values that become too specific to the dataset under study. The Dirichlet process-based CAT-inspired models tend to perform as well as the best finite mixture versions. As before, it may provide a more convenient random-effects method of capturing pattern-heterogeneity.

Table 3 summarizes the results across all single-gene alignments, again with cross-validation score relative to the GTR +Γ model. For finite mixture models, the results with 20 components are reported, which is where their performance was best. The same overall model rankings found with multigene alignments are found with all single-gene alignments. Note that a finite mixture with 20 components is a much richer model than traditional partitioning schemes used for protein-coding data, which are usually limited to three models, assigned to first, second, and third codon positions, respectively. This result suggests that pattern heterogeneity at the nucleotide level goes beyond the simple coding nature of the datasets.

**Table 3.** Cross-validation score relative the GTR + Γ with single-gene datasets.

	PB2	RAG1	RBCL	RHOD
F81	−3896.5 ± 228.9	−2010.6 ± 71.4	−263.2 ± 48.4	−290.4 ± 32.4
GTR	−1992.1 ± 144.1	−1295.4 ± 54.0	−188.2 ± 42.7	−251.4 ± 21.0
CAT${}_{f=20}$ Poisson	195.3 ± 55.3	161.9 ± 25.0	54.1 ± 40.2	48.1 ± 17.4
CAT${}_{f=20}$ GTR	381.7 ± 33.5	269.1 ± 20.5	56.7 ± 43.3	67.4 ± 13.4
CAT-Poisson	205.0 ± 55.1	165.1 ± 24.6	51.9 ± 38.6	47.7 ± 16.9
CAT-GTR	385.6 ± 31.6	272.9 ± 20.0	55.6 ± 40.8	67.1 ± 13.4
F81+Γ	−1944.6 ± 131.1	−738.1 ± 82.6	−72.8 ± 23.0	−40.6 ± 19.9
CAT${}_{f=20}$ Poisson + Γ	248.9 ± 50.4	150.8 ± 28.1	60.5 ± 42.9	63.3 ± 10.1
CAT${}_{f=20}$ GTR + Γ	420.7 ± 32.3	321.0 ± 27.4	66.7 ± 45.6	74.7 ± 7.0
CAT-Poisson + Γ	249.6 ± 49.6	192.6 ± 26.4	59.9 ± 45.7	60.7 ± 11.2
CAT-GTR + Γ	422.6 ± 33.0	322.5 ± 27.3	65.4 ± 45.0	74.5 ± 7.0

The relative contribution of each modeling approach again holds for single-gene datasets, as reported in Table 4. It is important to note that the cross-validation approach we utilize inherently tends to disfavor the richest models. This is because we are utilizing the cv-score as a measure of the predictive power of the model for the dataset as a whole, but we only present the model with four fifths of the data. Since the richest models may need more data to reliably infer parameters than simpler models, depriving them of the full dataset may have a greater impact on their performance. In other words, the true improvement in predictive power of the CAT-inspired models over homogeneous models is likely to be greater than what is reported herein.

**Table 4.** Pairwise cross-validation score differences with single-gene datasets.

Model difference	Models	PB2	RAG1	RBCL	RHOD
Mixture	F81 versus CAT-Poisson	4101.5	2175.7	315.04	338.1
Mixture	GTR versus CAT-GTR	2377.7	1568.3	243.8	318.6
Mixture	F81+Γ versus CAT-Poisson + Γ	2194.2	930.7	132.7	101.3
Mixture	GTR + Γ versus CAT-GTR + Γ	422.6	322.5	65.4	74.5
Γ distributed rates	GTR versus GTR + Γ	1992.1	1295.4	188.2	251.4
Γ distributed rates	F81 versus F81+Γ	1951.9	1272.5	190.4	249.9
Γ distributed rates	CAT-GTR versus CAT-GTR + Γ	37.0	49.7	9.8	7.3
Γ distributed rates	CAT-Poisson versus CAT-Poisson + Γ	44.5	27.5	8.0	13.0
Free exchangeabilities	F81+Γ versus GTR + Γ	1944.6	738.1	72.8	40.6
Free exchangeabilities	F81 versus GTR	1904.4	715.2	74.9	39.0
Free exchangeabilities	CAT-Poisson + Γ versus CAT-GTR + Γ	173.1	130.0	5.5	13.7
Free exchangeabilities	CAT-Poisson versus CAT-GTR	180.6	107.8	3.7	19.5

## 4 Conclusions and future directions

Early methods for comparing the predictive power of nucleotide substitution models were based in information-theoretic criteria. These criteria compared the likelihood scores obtained under a pair of models being compared, but also included a greater penalty on the higher dimensional model. This penalty was meant to ensure that the values of additional parameters were not too specific to the dataset under study, and would generalize well to other datasets. The main advantage of these criteria was their ease of use on small datasets. Cross-validation methods, on the other hand, require larger amounts of data; one must have data to spare (i.e. not used in model fitting) in order to actually test the predictive power of model on previously unseen data. Indeed, with our single-gene datasets, setting aside a fifth of the data leaves us with a partial gene on which to learn parameter values. The large error-bars displayed in Fig. 2 would likely prevent one from clearly distinguishing between some of the models of the GTR family using cross-validation approaches. However, methods based on cross-validation may be important in comparing models high in dimensionality (Liu *et al.* 2023). Future work should explore other cross-validation approaches, including the *leave-one-out* scheme.

While the ideas behind the nucleotide substitution modeling approaches studied herein have existed for decades, no careful measurements of their relative contribution to predictive power had yet to be conducted, to the best of our knowledge. Our study shows that modeling pattern heterogeneity is by far the most important contributor to a nucleotide substitution model’s performance, a result consistent with amino acid-level models (Bujaki and Rodrigue 2022). Of course, these models are parameter rich in comparison to single-matrix models, but this increased richness is accounted for intrinsically by the cross-validation framework.

Given these results, more in-depth studies on the various ways of modeling pattern heterogeneity seem warranted. Partitioning approaches may capture some of the pattern heterogeneity within a dataset, but they are limited to fixed-effect heterogeneity across partitions, which usually consist of genes, and/or codon positions. The random-effects mixture models studied here can capture more subtle variation across all positions at once. Still, quantifying the difference in performance between partitioning methods and the random-effects methods studied herein is pressing.

Other models that could be incorporated into the comparisons conducted here include: mixtures of nucleotide exchangeabilities (Pagel and Meade 2004), mixtures of gamma-distributions (Mayrose *et al.* 2005) or Dirichlet process approaches (Huelsenbeck and Suchard 2007) for a more subtle account of overall rate heterogeneity across sites, and models accommodating heterogeneity across the branches of the phylogeny (e.g. Blanquart and Lartillot 2006). The typical focus of such works was generally to show that the proposed modeling idea had merit over a null model that ignores the feature being captured. Such a focus is an important step in model development. We have emphasized here, however, that the larger model development program is greatly informed by quantitative comparisons of a new modeling idea relative to the broad classes of modeling approaches that are currently widely available.

Historically, most nucleotide-level phylogenetic analyses have utilized a protocol to select a substitution process from the GTR-family, along with an account of overall rates heterogeneity, while ignoring the pattern heterogeneity across gene alignments, or doing so in only a limited manner. The CAT-inspired nucleotide substitution models can flexibly accommodate pattern heterogeneity, at only a slightly increase computational cost. Thanks to the data-augmentation-based Markov chain Monte Carlo methods utilized in the PhyloBayes package (Lartillot 2006), the implementation of CAT-GTR +Γ requires only about 1% more computational time than GTR +Γ in the case of the largest dataset studied herein (Regier). We thus suggest that the CAT-inspired models be incorporated in model selection protocols given that their quantitative difference in predictive power largely overshadows any differences across GTR-family substitution matrices or gamma-distributed rates across sites.

## Data Availability

Data and software were all available prior to this particular work, and the text includes all references to their sources.
